# Knowledge discovery for Deep Phenotyping serious mental illness from Electronic Mental Health records

**DOI:** 10.12688/f1000research.13830.2

**Published:** 2018-05-08

**Authors:** Richard Jackson, Rashmi Patel, Sumithra Velupillai, George Gkotsis, David Hoyle, Robert Stewart

**Affiliations:** 1 Institute of Psychiatry, Psychology and Neuroscience, King's College London, London, SE5 8AF, UK; 2 South London and Maudsley NHS Foundation Trust, London, SE5 8AZ, UK; 3 School of Computer Science and Communication, TH Royal Institute of Technology, Stockholm, SE-100 44, Sweden; 4 Independent Researcher, Manchester, UK

**Keywords:** word2vec, natural language processing, serious mental illness, electronic health records, schizophrenia

## Abstract

**Background: **Deep Phenotyping is the precise and comprehensive analysis of phenotypic features in which the individual components of the phenotype are observed and described. In UK mental health clinical practice, most clinically relevant information is recorded as free text in the Electronic Health Record, and offers a granularity of information beyond what is expressed in most medical knowledge bases. The SNOMED CT nomenclature potentially offers the means to model such information at scale, yet given a sufficiently large body of clinical text collected over many years, it is difficult to identify the language that clinicians favour to express concepts.

**Methods:** By utilising a large corpus of healthcare data, we sought to make use of semantic modelling and clustering techniques to represent the relationship between the clinical vocabulary of internationally recognised SMI symptoms and the preferred language used by clinicians within a care setting. We explore how such models can be used for discovering novel vocabulary relevant to the task of phenotyping Serious Mental Illness (SMI) with only a small amount of prior knowledge.

**Results: **20 403 terms were derived and curated via a two stage methodology. The list was reduced to 557 putative concepts based on eliminating redundant information content. These were then organised into 9 distinct categories pertaining to different aspects of psychiatric assessment. 235 concepts were found to be expressions of putative clinical significance. Of these, 53 were identified having novel synonymy with existing SNOMED CT concepts. 106 had no mapping to SNOMED CT.

**Conclusions:** We demonstrate a scalable approach to discovering new concepts of SMI symptomatology based on real-world clinical observation. Such approaches may offer the opportunity to consider broader manifestations of SMI symptomatology than is typically assessed via current diagnostic frameworks, and create the potential for enhancing nomenclatures such as SNOMED CT based on real-world expressions.

## Introduction

The dramatic decrease of genetic sequencing costs, coupled with the growth of our understanding of the molecular basis of diseases, has led to the identification of increasingly granular subsets of disease populations that were once thought of as homogenous groups. As of 2010, the molecular basis for nearly 4 000 Mendelian disorders has been discovered ^1^, subsequently leading to the development of around 2 000 clinical genetic tests ^2^. The resulting ‘precision medicine’ paradigm has been touted as the logical evolution of evidence-based medicine.

Precision medicine has arisen in response to the fact that the real-world application of many treatments have a lower efficacy and a differential safety profile compared to clinical trials, most likely due to genetic and environmental differences in the disease population. Precision medicine seeks to obtain deeper genotypic and phenotypic knowledge of the disease population, in order to offer tailored care plans with evidence-based outcomes. Amongst the challenges presented by precision medicine is the requirement to obtain highly granular phenotypic knowledge that can adequately explain the variable manifestation of disease.

To realise the ambitions of precision medicine, large amounts of phenotypic data are required to provide sufficient statistical power in tightly defined patient cohorts (so called ‘Deep Phenotyping’ ^3^). Historical clinical data mined from Electronic Health Record (EHR) systems are frequently employed to meet the related use case of observational epidemiology. As such, EHRs are often posited as the means to provide extensive phenotypic information with a relatively low cost of collection ^4, 5^.

In order to standardise knowledge representation of clinically relevant entities and the relationships between them, phenotyping from EHRs often employs curated terminology systems, most commonly SNOMED CT. The use of such resources creates a common domain language in the clinical setting, theoretically allowing an unambiguous interpretation of events to be shared within and between healthcare organisations. The anticipated value of such a capability has prompted the UK National Information Board to recommend the adoption of SNOMED CT across all care settings by 2020 ^6^. However, the task of representing the sprawling and ever-changing landscape of healthcare in such a fashion has proven complex ^7– 10^. Although a complete description of the structure and challenges of SNOMED CT are beyond the scope of this paper, we describe how aspects of these problems manifest themselves in accordance with the task of phenotyping serious mental illness (SMI) from a real-world EHR system.

### Phenotyping SMI

The quest for empirically validated criteria for assessing the symptomatology of mental illness has been a long term goal of evidence-based psychiatry. SMI is a commonly used umbrella term to denote the controversial diagnoses of schizophrenia (encoded in SNOMED as SCTID: 58214004), bipolar disorder (SCTID: 13746004), and schizoaffective disorder (SCTID: 68890003). While field trials of DSM-5 have revealed promising progress in reliably delineating these three conditions in clinical assessment ^11^, such diagnostic entities continue to have low clinical utility ^12– 14^. Recent evidence from genome-wide association studies appears to suggest that such disorders share common genetic loci, further countering the argument that SMI can be classified into discrete, high level diagnostic units ^15^. In terms of clinical practice, the presenting symptomatology of SMI is usually the basis for treatment. This is often characterised by abnormalities in various mental processes, which are in turn categorised according to broad groupings of clinically observable behaviours. For instance, ‘positive symptoms’ refer to the presence of behaviours not seen in unaffected individuals, such as hallucinations, delusional thinking and disorganised speech. Conversely, ‘negative symptoms’, such as poverty of speech and social withdrawal refer to the absence of normal behaviours. Such symptomatology assessments are organised via an appropriate framework such as Postive and Negative Symptom Scale ^16^ (PANSS) or Brief Negative Symptom Scale ^17^. Accordingly, SNOMED CT includes coverage for many of these symptoms, generally within the ‘Behaviour finding’ branch (SCTID: 844005).

A qualifying factor regarding the adoption of SNOMED amongst SMI specialists might therefore require that the list of clinical ‘finding’ entities in SNOMED are sufficiently expansive and diverse to represent their own experiences during patient interactions. Specifically, this may manifest as two key challenges for terminology developers.

First, insight must be obtained regarding real-world language usage such that universally understood medical concepts, encompassing hypernomy, synonymy and hyponomy. Similarly, the abundant use of acronyms in the medical domain means that a large percentage of acronyms to have two or more meanings ^18^, creating word sense disambiguation problems. As such, significant efforts have arisen to supplement these types of knowledge bases with appropriate real-world synonym usage extracted from EHR datasets ^19^. The problem may be considered analogous to difficulties in the recognition, classification and mapping of technical terminology variants throughout the biomedical literature, which is known to be an impediment to the construction of knowledge representation systems (see 20 for a review).

Second, if there is controversy over international consensus in a particular area of medicine, the use of ‘global’ perspectives may not be sufficient to meet local reporting/investigatory requirements. Such issues are particularly pertinent in mental health where many diseases defy precise definition and biomarker development has yielded few successes ^21^. More generally, all medical knowledge bases are incomplete to one degree or another. The opportunity to utilise large amounts of EHR data to discover novel observations and relationships arising from real-world clinical practise must not be overlooked.

Given a sufficiently large corpus of documents, typically written by hundreds of clinical staff over several years, it is often difficult to track the evolution of vocabulary used within the local EHR setting to describe potentially important clinical constructs. In previous work, we describe our attempts to extract fifty well known SMI symptomatology concepts from a large electronic mental health database resource ^22^, based upon the contents of such frameworks. During the course of manually reviewing clinical text, we made two subjective observations of the documentation resulting from clinician/patient interactions:

- The tendency of clinicians to use non-technical vocabulary in describing their observations
- The occasional appearance of highly detailed, novel observations that do not readily fit into known symptomatology frameworks

Such observations may feasibly have clinical relevance, for example, as non-specific symptomatology prodromes ^23^. On the basis that the modelling of SMI for precision medicine approaches require the full dimensionality of the disease to be considered, we sought to explore these observations further.

In this study, we present our efforts to utilise *a priori* knowledge discovery methods to identify preferences in real-world language usage that reflect clinically relevant SMI symptomatology within the context of a large mental healthcare provider. We contrast and compare these patterns with a modern version of the UK SNOMED CT (v1.33.2), and suggest how such approaches may offer novel and/or more granular symptom expressions from patient/clinician interactions when used to supplement resources such as SNOMED CT, potentially offering alternatives to classify psychiatric disorders with finer resolution and greater real-world validity.

## Methods

**Figure 1.**
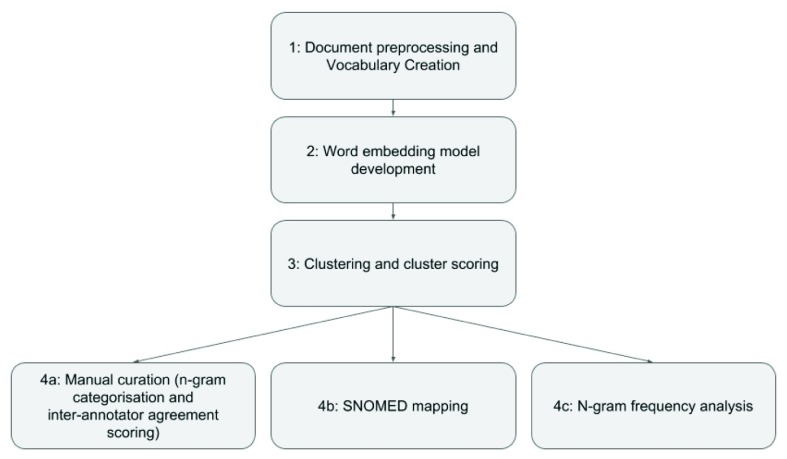
Overview of project workflow.

Our general approach for SMI knowledge discovery is composed of several discrete steps. An overview of the workflow is given in Figure 1.

### Corpus creation from the Clinical Record Interactive Search

The South London and Maudsley NHS Foundation Trust (SLaM) provides mental health services to 1.2 million residents over four south London boroughs (Lambeth, Southwark, Lewisham and Croydon). Since 2007, the Clinical Record Interactive Search (CRIS) ^24^ infrastructure programme has been operating to offer a pseudonymised and de-identified research database of SLaM’s EHR system. As the CRIS resource received ethical approval as a pseudonymised and de-identified data source by Oxford Research Ethics Committee (reference 08/H0606/71+5), patient consent was not required for this study.

11 745 094 clinical documents were collected from the CRIS database from the period 01/01/2007 - 27/10/2016 on the basis that the 20 472 associated patients were assigned an SMI ICD10 code of F20, F25, F30 or F31 at some point during their care, in accordance with current clinical practice.

### Pre-processing and vocabulary creation

Sentences and tokens were extracted from each document using the English Punkt tokeniser from the NLTK 3.0 suite ^25^. Each token was converted to lower case. A vocabulary was then constructed of all 1-gram types in the corpus, supplemented with frequently occuring bi-grams and tri-grams using the Gensim ^26^ suite and the sampling method proposed by Mikolov *et al.* ^27^. Bi-grams and tri-grams with a minimum frequency of 10 occurrences in the entire corpus were retained, to give a total vocabulary size of 896 195 terms (617 095 unigrams, 277 490 bigrams, 303 trigrams and 1 307 non-word entities). No further assumptions about the structure of the data, such as the need for stemming/lemmatisation, were made.

### Building a word embedding model

The distributional hypothesis was first explored by Harris ^28^, which proposed that, given a sufficiently large body of text, linguistic units that co-occur in the same context are likely to have a semantically related meaning. Modelling the distribution of such units may therefore have value for a wide range of natural language processing applications. Models of distributional semantics, including word embeddings, are techniques that aim to derive models of semantically similar units in a corpus of text by co-locating them in vector space. In recent years, the use of the Continuous Bag-of-Words (CBOW) model proposed by Mikolov *et al.* ^29^ has risen to prominence, owing to its ability to accurately capture semantic relationships whilst scaling to large corpora of text ^27^. Recently, the CBOW model has been used to identify the semantic similarities between single word entities in biomedical literature and clinical text ^30^, suggesting that biomedical literature may serve as a useful proxy for clinical text, for tasks such as synonym identification and word sense disambiguation tasks under limited conditions ^30^.

A full description of the CBOW architecture is discussed in 31. For brevity, we describe only the key features used in our work here. The purpose of the architecture is to ’learn’ in an unsupervised manner, a representation of the semantics of different terms, given an input set of documents. CBOW might be described as a simple feed forward neural network consisting of three layers. An input layer *X* composed of *o* nodes (where *o* is the number of unique terms in a corpus produced from our above described pre-processing), a hidden layer *H* of a user defined size *n* (usually between 100 and 300), and an output layer *Y* that is also composed of *o* nodes. Every node in *X* is connected to every node in *H*, and every node in *H* is connected to every node in *Y* . Between each of the layers is a matrix of weight values; for the *X* and *H* layer, an ‘input’ matrix of dimensions *o × n* (hereafter denoted *W*); and between the *H* and the *Y* layer, an ‘output’ matrix of dimensions *n × o* (denoted *W*′). The output of training the neural network is to produce weights in each of these matrices. The weights learnt in the *W* matrix might be intuitively described as the semantic relationships between each term in the vocabulary as represented in vector space, with semantically similar words located in closer proximity to each other. Weights in the *W*′ matrix represent the predictive model from the *H* to the *Y* layer. A training instance is composed of a group of terms, known as a context. A context can be composed of natural language structures, such as sentences in a document, or more complex arrangements, such as a sliding window of terms (usually between 5 and 10) that move over each token in a document (potentially ignoring natural grammatical structures). For a given input term, the input into the nodes on the hidden layer is the product of each vector index in matrix *W* corresponding to each context word and the average vector. From the *H* to the *Y* layer, it is then possible to score each term using the *W*′ matrix, from which a posterior probability is obtained for each word in the vocabulary using the softmax function. The weights in each matrix are then updated using computationally efficient hierarchical softmax or negative sampling approaches. Once training is complete, the semantic similarity of terms is often measured via their cosine distance between vectors in the *W* matrix.

Using the Gensim implementation of CBOW and our previously constructed vocabulary, we trained a word embedding model of *n* = 100 over our SMI corpus to produce a vector space representation of our clinical vocabulary. Due to patient confidentiality, offline access to records was not feasible and so only a limited number of epochs of training could be performed. However, due to the relatively narrow/controlled vocabulary employed in clinical records (compared to normal speech/text) the range of possible input vectors was narrower than might otherwise be expected, and even a single epoch of training appeared to yield meaningful clusters that could be identified with SMI. As we were primarily intending to identify initial clusters for validation by clinical experts it was felt that single epoch of training, over the 20M clinical records available, was sufficient.

### Vocabulary clustering and cluster scoring

The task of clustering seeks to group similar dataset objects together in meaningful ways. In unsupervised clustering, the definition of ‘meaningfulness’ is often subjectively defined by the human observers. In our task, we sought to identify clusters of terms derived from our word embedding model that represent semantically linked components of our clinical vocabulary, based on the theory that our word embedding model would cause related symptom concepts to appear close to each other within the vector space.

A particular challenge in the development of clustering algorithms is achieving scalability to large datasets. Since many clustering algorithms make use of the pairwise distance between *n* samples (or terms, in our case), the memory requirements of such algorithms tend to run in the order of *n* ^2^. One such algorithm that does not suffer from this limitation is *k*-means clustering. *k*-means clustering is a partitional clustering algorithm that seeks to assign *n* samples into a user defined *k* clusters by minimising the squared error between each centroid of a cluster and its surrounding points. A global (although not necessarily optimal) solution is derived when the algorithm has minimised the sum of squared errors across all *k* clusters, subject to some improvement threshold or other stopping criteria. For all experiments, we used the *k*-means++ implementation from the Scikit-Learn framework ^32^ with 8 runs each time, to control against centroids emerging in local minima.

The key parameter for *k*-means clustering is the selection of *k*. While techniques exist for estimating an appropriate value, such as silhouette analysis and the ‘elbow method’ ^33^, these utilise pairwise distances between samples, creating substantial technical limitations for large matrices in terms of memory usage. To overcome this, we opted for a memory efficient version of the elbow method, involving plotting the minimum centroid distance for different values of *k*. The intuition behind this approach is that every increase in *k* is likely to result in a smaller minimum centroid distance in vector space (subject to a random seed for the algorithm). As *k* increases, genuine clusters should be separated by a steady decline in minimum centroid distance. However, when the slope of the decline flattens out (i.e. the ‘elbow’ of the curve), assignment of samples to new clusters is likely to be random).

With the data clustered, we sought to identify one or more clusters of interest for further examination. To this end, we devised a simple ‘relevance’ cluster scoring approach based upon prior knowledge of common SMI symptom concepts. The intuition behind our approach is that the training of the Word2Vec model will cause terms that represent ‘known’ concepts of SMI symptomatology to colocate in close proximity to each other in vector space, and the clustering approach will place them in the same cluster, along with other terms that theoretically relate to these SMI symptomatology concepts. The additional contents of this cluster may therefore hold terms that represent concepts of SMI symptomatology undefined by our team, but in natural use by the wider clinical staff of the SLaM Trust during the course of their duties. By identifying the richest cluster(s) in terms of the known SMI symptomatology lexicon, we sought to drastically reduce the search space of terms in the corpus to carry forward for human assessment.

We selected 38 internationally recognised symptom concepts of SMI based upon their expression in SMI frameworks and on their specificity in clinical use ( Table 1), to form the basis of our scoring algorithm. For instance, we did not select ‘loosening of associations’, due to the different word sense that the word ‘associations’ appears in, such as ‘housing associations’, and organisational references such as ‘Stroke Association’. Rather, we chose symptoms such as ‘aggression’, ‘apathy’ and ‘agitation’, which are less likely to have different word sense interpretations in the context of SMI clinical documents.

**Table 1.** Known symptomatology concepts and Prior Concept vocabulary matching sequences used for cluster scoring. An underscore represents a bigram match.

SMI symptom	Prior Concept matching character sequence
aggression	aggress
agitation	agitat
anhedonia	anhedon
apathy	apath
affect	affect
catalepsy	catalep
catatonic	cataton
circumstantial	circumstant
concrete	concrete
delusional	delusion
derailment	derail
eye contact	eye_contact
echolalia	echola
echopraxia	echopra
elation	elat
euphoria	euphor
flight of ideas	foi
thought disorder	thought_disorder
grandiosity	grandios
hallucinations	hallucinat
hostility	hostil
immobility	immobil
insomnia	insomn
irritability	irritab
coherence	coheren
mannerisms	mannerism
mutism	mute
paranoia	paranoi
persecution	persecut
motivation	motivat
rapport	rapport
posturing	postur
rigidity	rigid
stereotypy	stereotyp
stupor	stupor
tangential	tangenti
thought block	thought_block
waxy	waxy

For each of the 38 concepts, we produced a set of terms constituting stems and appropriate synonyms/acronyms as described in Table 1, in order to produce a set of character sequences representing existing domain knowledge, or ‘prior concepts’ (hereafter, termed PCs) that could be matched against each term in each cluster via regular expressions. With this matching criterion, we scored each cluster based on the number of hits to derive a cluster/PC count matrix *x* where *x*_*i*, *j*_ represents the count of the *i*th PC in the *j*th cluster. For example, a cluster containing the 1-gram ‘insomnia’ and ‘insomniac’ would receive a count of two for the ‘insomni’ PC. For each PC, we then calculated a vector of the minimum count per concept across all clusters:

$$u_{i}={min}_{j\in J}x_{ij,}i=1,\ldots m.\;(1)$$

where *m* is 38 (denoting the number of PCs we describe in Table 1). Similarly, we generated a vector of maximum count per PC across all clusters:

$$v_{i}={max}_{j\in J}x_{ij,}i=1,\ldots m.\;(2)$$

to enable us to rescale the value of each PC/cluster count to between 0 and 1 into a matrix *x*′:

$${x^{\prime }}_{ij}=\frac{x_{i,j}-u_{i}}{v_{i}-u_{i}}\;(3)$$

The purpose of rescaling in such a way was to prevent overrepresented PCs unduly influencing the overall result (for instance, a PC with many hits in a cluster would unduly bias the score towards that concept, whereas we sought a scoring mechanism that would weigh all input PCs equally, regardless of their frequency).

Finally, we summed all rescaled PC counts per cluster, and divided by the total cluster size to provide a score per cluster *z* representing the value of the:

$$z_{j}=\frac{\sum_{i=1}^{m}{x^{\prime }}_{ij}}{s_{j}}\;(4)$$

where *s* is a vector of the total count of terms in each cluster. The purpose of dividing by cluster size was to prevent the tendency of larger clusters to score higher on account of their size.

To select clusters for further investigation, the robust median absolute deviation (MAD) statistic was chosen (the distribution of our cluster scores was non-normal). This precipitated clusters that were the most valuable, in terms of the breadth of PC concept hits they contain. We adopted a conservative approach to cluster selection by choosing clusters that scored at least six MAD above the median score for further processing, which is approximately equivalent to four standard deviations for a normally distributed dataset.

We provide a worked example of this technique in the code repository that accompanies this paper, using publically available data.

### Expert curation of symptom concepts, frequency analysis and SNOMED CT mapping

The contents of the top scoring clusters underwent a two stage curation process. The first stage was performed by an informatician, and involved several simple string processing tasks to filter out uninteresting terms. Such processes included removal of terms that contained tokenisation failures (for example, single character non-word tokens such as ‘y’, ‘p’) and other constructs that had low information content, such as terms composed of stop words. A final manual check followed to reduce the annotator burden required by the clinical team.

The second, more important, stage was composed of independent annotation of the curated concept list by two psychiatrists, to identify likely synonyms and new symptomatology based on their clinical experience. Each concept was assigned to one of the below 8 ‘substantive’ categories, or a 9th ‘other’ category. The categories were derived from 34, and the experience of the team Clinical Psychiatrists.

**Appearance/Behaviour** Implying a real-time description of the way a patient appears or behaves (including their interaction with the recording clinician)
**Speech** Anything implying a description of any vocalisation (i.e. theoretically a subset of behaviour but restricted to vocalisations)
**Affect/Mood** Implying clinician-observed mood/emotional state (i.e. theoretically a subset of appearance but restricted to observed emotion), or implying self-reported mood/emotional state (i.e. has to imply a description that a patient would make of their own mood; theoretically a subset of thought)
**Thought** Implying any other thought content
**Perception** Implying any described perception
**Cognition** Implying anything relating to the patient’s cognitive function
**Insight** Implying anything relating to insight (awareness of health state)
**Personality** Anything implying a personality trait or attitude (i.e. something more long-standing than an observed behaviour at interview)
**Other** A mixed bag of definable terms that do not fit into the above. Common examples included anything implying information that will have been collected as part of a patient’s history, often of behaviours that would have to have been reported as occurring in the past and cannot have been observed at interview, but also which cannot be termed a personality trait. Alternatively, anything where insufficient context was available to make a decision

Inter annotator agreement (IAA) was measured with the Cohen’s Kappa agreement statistic ^35^.

To explore the frequency of both our prior symptomatology concepts and the newly curated ones in our symptom clusters, we counted the number of unique patient records and the number of unique documents in which the stems of each term appeared. To protect patient anonymity, we discarded any concept that appeared in ten or fewer unique patient records. Finally, we mapped the remaining concepts to SNOMED CT, UK version v1.33.2, using the following method. First, the root mopheme of each concept was matched to a relevant finding, observable entity or disorder type in SNOMED CT. If a match could not be found, SNOMED CT was explored for potential synonymy, or other partial match. If a clear synonym could not be found, we classified the concept as novel.

## Results

### Word embedding model training

Processing the corpus of SMI clinical documents took approximately 100 hours on an 8-core commodity hardware server. Documents were fed sequentially from an SQL Server 2008 database operating as a shared resource, with an additional overhead likely resulting from network latency.

### Parameter selection for *k*-means clustering

**Figure 2.**
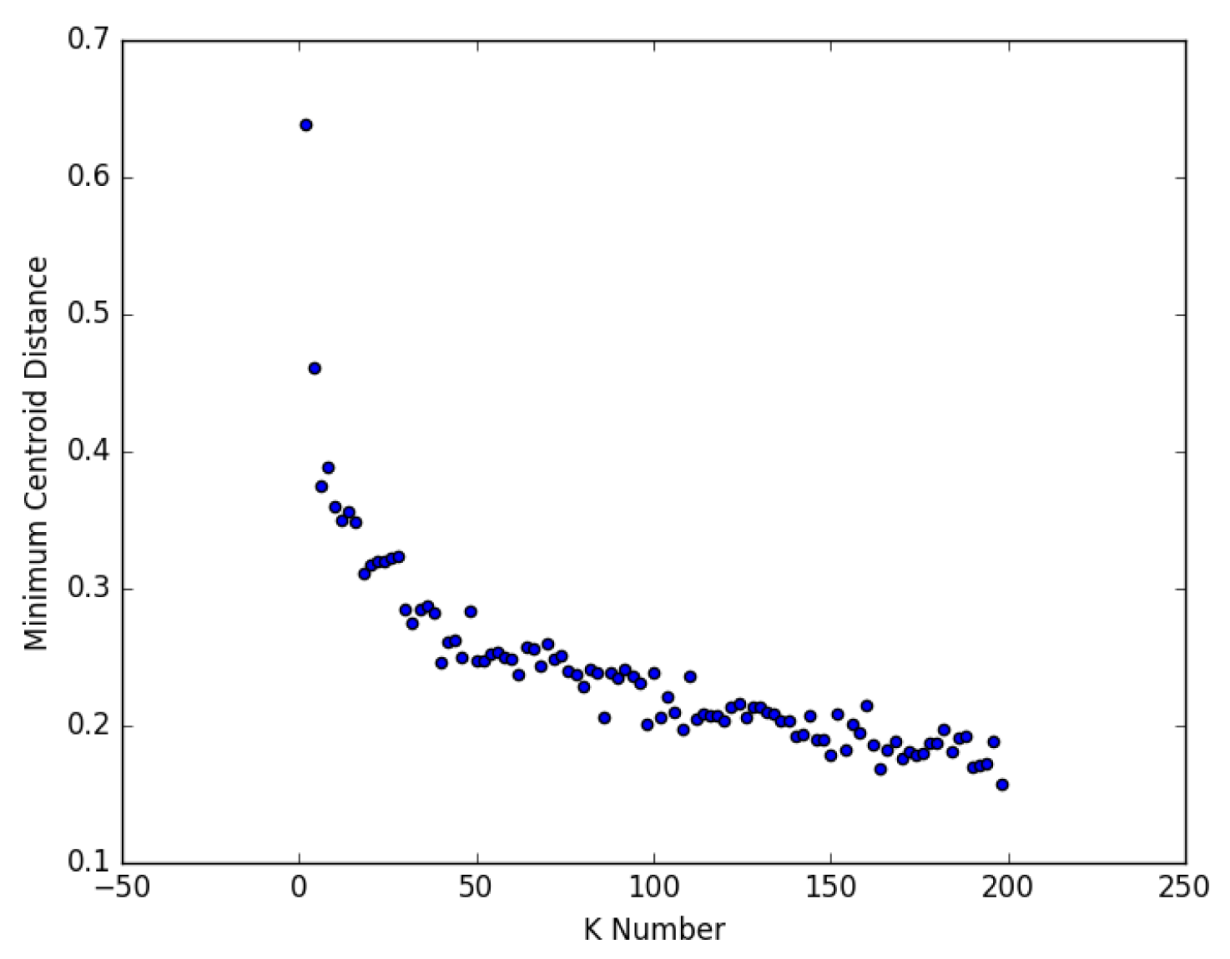
Selecting K for K-means++.

Figure 2 shows a scatterplot of variable values of *k* and the resulting minimum centroid distance. This suggests a *k* value of around 50–75 may be optimal for our data. On this basis, we chose a *k* value of 75.

### Cluster scoring

**Figure 3.**
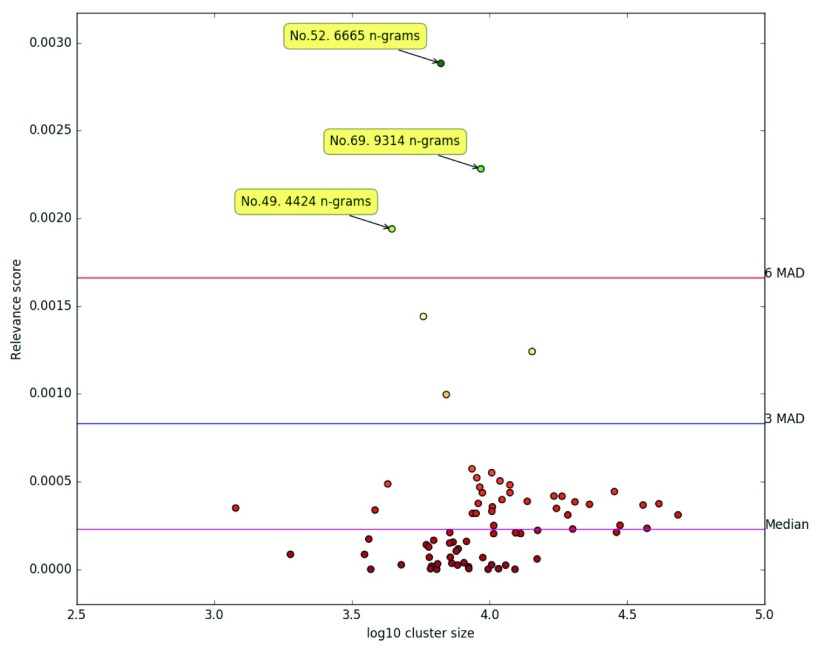
Scoring of clusters according to known symptomatology content. Each dot represents a unique cluster. The unique cluster IDs of the most relevant clusters according to our scoring algorithm are labelled.

The application of our relevancy scoring algorithm to the 75 derived clusters resulted in a median score was 0.000229 and a MAD of 0.000277, and is visualised in Figure 3.

Three clusters emerged with a score at least six MADs outside of the median cluster score: No. 52 (score: 0.002883), containing 6 665 terms, No. 69, containing 9 314 (score: 0.002282) terms and No. 49 (score: 0.001940), containing 4 424 terms. Taken together, these three clusters contained a total of 20 403 terms.

### Expert curation of symptom concepts, frequency analysis and SNOMED CT mapping

The combined 20 403 terms were taken forward for curation as described above. The first phase of curation reduced the list to 519 putative concepts. The majority of eliminated terms were morphological variations, misspellings and tokenisation anomalies of singular concepts. For instance, 84 variations were detected for the stem ‘irrit*’ (as in ‘irritable’). Other terms were removed because insufficient context was available for a reasonable clinical interpretation, such as ‘fundamentally unchanged’, ’amusing’ and ‘formally tested’. Finally, terms that appeared to have no relevance to symptomatology at all were removed, such as dates and clinician names.

Expert curation by two psychiatrists of the 557 concepts (519 discovered concepts and 38 prior concepts) produced a Cohen’s Kappa agreement score of 0.45, where 337 concepts were assigned to one of our 9 categories independently by expert psychiatric curation. Of the 337 concepts, 235 were assigned to a substantive category (i.e. not the indeterminate ‘other’ group). Table 2 shows the number of terms per category where agreement was reached.

**Table 2.** Counts of terms where annotators independently agreed by category.

Category	Count
Affect/Mood	6
Appearance/Behaviour	78
Cognition	6
Insight	2
Mood/Anxiety/Affect	26
Other	102
Perception	9
Personality	23
Speech	63
Thought	22

Supplementary File 1 is a CSV table of all 557 terms. In addition to the term itself, the table contains the following information; the counts of the unique patient records of our 20 472 patient SMI cohort in which the term was detected; the counts of the unique documents of the 11 745 094 clinical document corpus wherein the term was detected; the category assigned to the term by each of our clinical annotators, and the SNOMED CT ID code for each term, where mapping was possible.

The most frequently detected concept mentions include ‘affect’ (detected in 91% of patients), ‘eye contact’ (85%), ‘hallucinations’ (85%), ’delusions’ (83%) and ‘rapport’ (81%). Other concepts follow a long tailed distribution, with mentions of the top 407 concepts found in at least 100 unique patient records.

Regarding SNOMED CT mapping, it was possible to suggest direct mappings for 177 concepts and to suggest synonymy or partial mapping for another 53 concepts. This left a remaining 327 concepts that did not appear to be referenced in SNOMED CT, of which 106 were classified as belonging to a substantive symptom category by independent curation.

**Figure 4.**
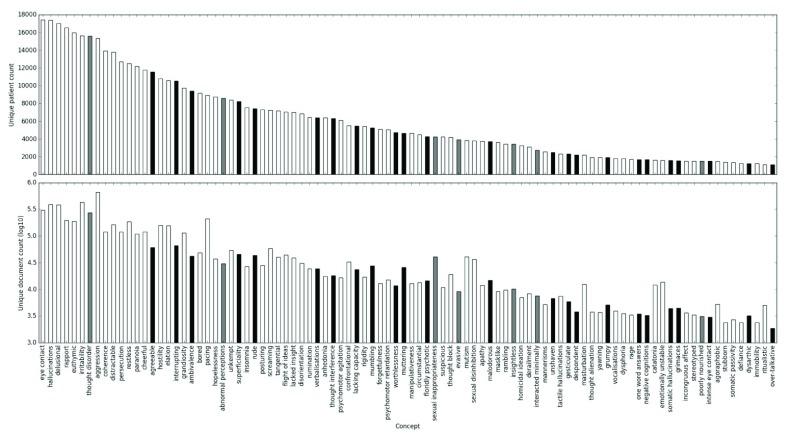
Frequency of terms across all SMI documents in CRIS. White bars represent concepts that were found to exist in SNOMED CT. Grey bars represent partial/uncertain matches, or novel synonyms of existing SNOMED CT concepts. Black bars represent concepts with no SNOMED mapping.

Figure 4 visualises the top 20% most frequent terms by appearance in unique patient records, where annotators agreed and were not classified as our ‘other’ grouping.

Owing to the difficulty of the IAA and categorisation task, an extended analysis of the top 40% most frequent terms by appearance in unique patient records, irrespective of IAA and categorisation is provided in Supplementary Figure 1.

In this project, we sought to explore SMI symptomatology and other language constructs as expressed by clinicians in their own words, using more than ten years of observations made during real-world clinician/patient interactions from more than 20 000 unique SMI cases. Within the context of a large mental healthcare provider, the results of our vocabulary curation efforts suggest that psychiatrists make use of a wide range of vocabulary to describe detailed symptomatic observations.

Many of the curated entities where both annotators agreed upon a substantive category map directly to preferred terms or synonyms of well known symptomatology constructs as described in SNOMED CT. Reassuringly, many of most frequently encountered entities as represented by unique patient count are represented in SNOMED CT, suggesting that SNOMED CT offers a reasonable coverage of what clinicians deem to be the most salient features of a psychiatric examination.

Nevertheless, our work produces evidence to suggest that many suitable synonyms are currently missing from SNOMED CT symptom entities. For instance, ‘aggression’ is commonly observed in SMI patients. Our results indicate that this construct might also be referred to by adjectives and phrases such as ‘combatative’ [ *sic*], ‘assaultative’ [ *sic*], ‘truculent’, ‘stared intimidatingly’ and ‘stared menacingly’, amongst others. Similarly, direct synonyms of ‘paranoia’ might include ‘suspiciousness’, ‘mistrustful’ and ‘conspirational’[ *sic*].

In addition, many of the curated constructs appear to reflect more granular observations of known symptomatology. For example, the PANSS utilises a 30-point scale of different symptomatology constructs. Specifically regarding abnormal speech, the PANSS provide guidance amounting to the high level clinical scrutiny of ‘lack of spontaneity & flow of conversation’. However, clinical expressions of speech within our dataset suggest around 68 distinct states, including ‘making animal noises’, ‘staccato quality’, ‘easily interruptible’, ‘prosody’ and ‘silently mouthing’.

We note the occurrence of several constructs that defy classification under existing schemas of SMI symptomatology, such as behaviours of ‘over politeness’, ‘over complimentary’, ‘spending recklessly’ and ‘shadow boxing’. The clinical interpretation of such entities is a non-trivial exercise, and is out of scope for this piece. Nevertheless, word embedding models may offer the potential to gain insight into potentially novel symptomatology constructs observed from real-world clinician/patient interactions. Future work might explore the context for such constructs in more detail.

The emergence of such diverse language in turn has implications for how SNOMED CT might be implemented within an SMI context, raising the question of whether such gaps represent significant barriers to the use of SNOMED CT as a phenotyping resource. The issue of SNOMED CT’s sufficiency in this context has previously been raised for other areas, such as rare disease ^36^, psychological assessment instruments ^37^ and histopathology findings ^38^. However, in fairness, SNOMED CT is not a static resource, but an international effort dependent on the contributions of researchers. Perhaps a more pertinent question for the future development of SNOMED CT concerns balancing its objective to be a comprehensive terminology of clinical language (capable of facilitating interoperability and modelling deep phenotypes within disparate healthcare organisations across the globe) and the overwhelming complexity it would need to encompass in order to not constrain its users. Certainly, at more than 300 000 entities in its current incarnation, its size already presents problems in biomedical applications ^39^.

### Limitations and future work

On the basis that manifestations of symptoms are the result of abnormal mental processes, novel symptom entities possibly represent observations of clinical significance. However, one particular complication in validating the clinical utility of novel symptomatology constructs with historic routinely recorded notes arises from systemic biases in EHR data. Specifically, the breadth and depth of symptomatic reporting is likely to be highly variable for a number of reasons. For instance, established symptoms as defined by current diagnostic frameworks are likely to be preferentially recorded, as clinicians are mandated to capture such entities in their assessments. On the other hand, constructs that fall outside of such frameworks may only be recorded as tangential observations made during patient/clinician interactions. Regardless of whether they are observed or not, without an established precedent of their clinical utility, they may be subject to random variation as to whether they are documented in a patient’s notes. This is borne out by the tendency of SNOMED CT-ratified concepts to appear more frequently in unique documents compared to our derived expressions. The validation of new symptoms from historic data is therefore something of a ‘chicken and egg’ situation, a widely-discussed limitation of the reuse of EHR data ^40, 41^. Nevertheless, our frequency analysis of our discovered constructs suggests that there is evidence that many are observed often enough to warrant their consideration within an expanded framework. Similarly, older frameworks with a limited scope of symptomatic expression were likely designed with pragmatic constraints around speed and reproducibility of assessment in mind. However, modern technology allows for a far greater scope of data capture and validation going forward, creating opportunities to develop new frameworks that maximise the value of psychiatric assessment. Future work in this domain might seek statistical validation via randomised experimental design, as opposed to observational study.

Our work suggests an approximate correlation between patient and document count, such that intra and inter patient symptomatological clinical language usage varies relatively consistently. However, some notable exceptions to this correlation (i.e. with a higher document level frequency to patient record level frequency) include ‘aggression’, ‘pacing’, ‘sexual inappropriateness’, ‘sexual disinhibition’ and ‘mutism’. Further work might seek to study these effects in greater detail, to uncover whether they represent a systemic bias in how such concepts are represented in the EHR.

The results of our IAA exercise between two experienced psychiatrists suggested a moderate level of agreement in categorising the newly identified constructs. Given that this annotation exercise did not provide any context beyond the term, and that the nature of SMI symptom observation is somewhat subjective, perhaps it is to be expected that agreement was not higher. As suggested during peer review, providing a concordance of some of the instances of each term, along with expert panel discussion and engagement with international collaborative efforts in SMI research may prove valuable in seeking more formal definitions of the identified concepts.

Our method for vocabulary building produced nearly 1 million terms. A manual annotation of this list may have resulted in further discoveries, although would have been intractable in practical terms. To reduce the volume of terms taken forward for curation, we employed a word embedding model with a clustering algorithm. With our cluster scoring methodology that makes use of existing domain knowledge, we were able to successfully produce meaningful clusters of terms reflecting the semantics of SMI symptomatology. However, as with many unsupervised tasks, it is difficult to determine whether an optimal solution has been achieved. In particular, the emergence of three ‘symptom’ clusters instead of one indicates sub-optimal localisation of symptom constructs in vector space. Addressing such a problem is multifaceted. For technical reasons, only a single epoch of training was possible in this exercise. Additional epochs would likely contribute to better cluster definition, in turn allowing us to reduce the value of our *k* parameter. In addition, spell checking and collapsing terms into their root forms may also have assisted. However, the latter may have also created new word sense disambiguation problems if common, symptom-like morphemes also appear in nonsymptomatological assessment contexts.

After clustering, a two stage manual curation of more than 20 000 terms was necessary. Methods that produce a smaller vocabulary might conceivably reduce annotator burden. This might include the use of spell checkers and stemming/lemmatisation to correct and normalise tokens, at the risk of introducing new issues associated with morphological forms in word embedding model building. For this attempt, we took the conscious decision to make as few assumptions about the underlying structure of the data as possible.

During peer review, it was suggested that recent advancements in topic modelling approaches may be relevant to our work. Many groups have sought to combine the popular technique of Latent Dirichlet Allocation (LDA) ^42^ with word embedding models to derive appropriate terminology for a given topic ^43– 45^. For instance, Nguyen *et al.* ^46^ propose an extension of LDA that makes use of a word embedding model trained on a very large corpus of text to improve the performance of topic coherence modelling on several datasets. Future work might seek to explore such techniques, and (assuming regulatory barriers can be overcome), the potential of creating word embedding models from very large clinical text corpora by combining data with other care organisations.

## Conclusions

Evidence-based mental health has long sought to produce disease model definitions that are both valid, in the sense they represent useful clinical representations that can inform treatment, and reliable, in that they can be consistently applied by different clinicians to achieve the same outcomes. In practice this has proven difficult, due to the often subjective nature of psychiatric examination/phenotyping and insufficient knowledge about the underlying mechanisms of disorders such as SMI. Here, we demonstrate that clinical staff make use of a diverse vocabulary in the course of their interactions with patients. This vocabulary often references findings that are not represented in SNOMED CT, raising questions about whether clinicians should observe the constraints of SNOMED CT or whether SNOMED CT should incorporate greater flexibility to reflect the nature of mental health. It is outside the scope of this work to explore how the granularity of symptom-based phenotyping affects patient outcomes, although the possibility of offering a fully realised picture of symptom manifestation may prove valuable in future endeavours of precision medicine.

## Data availability

The data referenced by this article are under copyright with the following copyright statement: Copyright: © 2018 Jackson R et al.

Data associated with the article are available under the terms of the Creative Commons Zero "No rights reserved" data waiver (CC0 1.0 Public domain dedication). 

The CRIS dataset is a pseydonymised and de-identified case registrar of electronic health records of the SLaM NHS Trust. It operates under a security model that does not allow for open publication of raw data. However, access can be granted for research use cases under a patient-led security model. For further information and details on the application process, please contact cris.administrator@kcl.ac.uk or visit the website: https://www.maudsleybrc.nihr.ac.uk/facilities/clinical-record-interactive-search-cris/. Alternatively, you may write to the CRIS team at: 

PO Box 92 Institute of Psychiatry, Psychology & Neuroscience at King’s College London 16 De Crespigny Park London SE5 8AF

Example code used in this analysis is available at: https://github.com/RichJackson/clustering_w2v

## Supplementary material

**Supplementary File 1:** This file contains all of the 557 terms taken forward for expert annotation. It includes SNOMED mappings where possible, unique document and patient counts within the corpus, and the annotations provided by RP and RS.

**Supplementary Figure 1:** This file is an expanded visualisation of the frequency analysis figure contained in the main manuscript, with the agreement and nonsubstantive ‘other’ classification restrictions lifted.
