# Supplementary figures and images for: Knowledge discovery for Deep Phenotyping serious mental illness from Electronic Mental Health records
Source: F1000Res. 2018 May 8;7:210. Originally published 2018 Feb 21. [Version 2] doi: 10.12688/f1000research.13830.2 (PMC5968362; doi:10.12688/f1000research.13830.2)

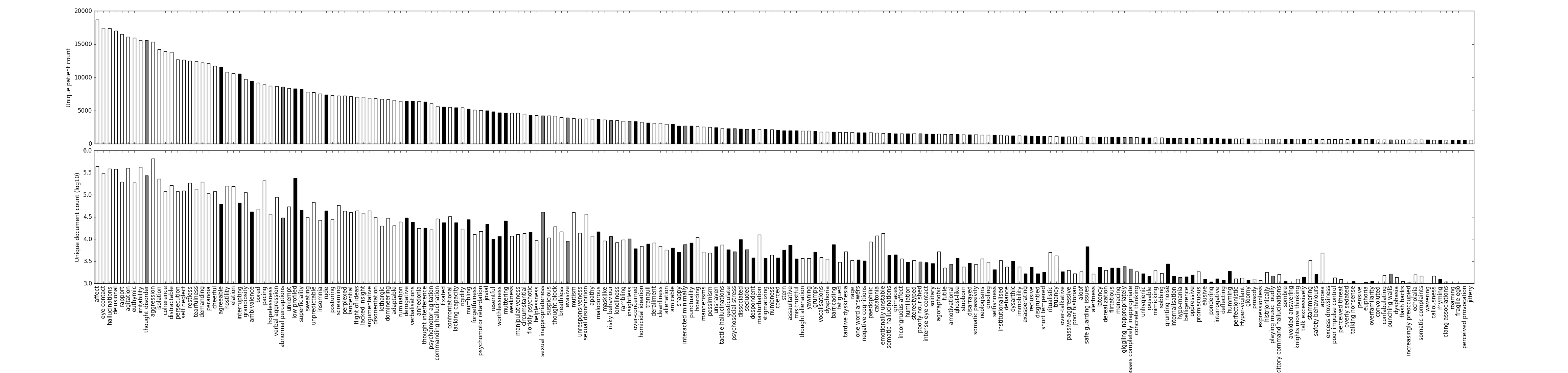

Supplement: Supplementary file 2 [file f1000research-7-16078-s0001.tgz › 3df4efde-6d75-4f7a-b058-5c9937ee5446.png]
