# Supplementary material for: Knowledge discovery for Deep Phenotyping serious mental illness from Electronic Mental Health records
Source: F1000Res. 2018 May 8;7:210. Originally published 2018 Feb 21. [Version 2] doi: 10.12688/f1000research.13830.2 (PMC5968362; doi:10.12688/f1000research.13830.2)
Supplement: Peer review history

# Peer review: Referee response for version 2

**DOI:** 10.5256/f1000research.16078.r33789

**Published:** 2018-05-24

**Authors:** Karin Verspoor

**Affiliations:** 1 School of Computing and Information Systems, The University of Melbourne, Melbourne, VIC, Australia; 2 Health and Biomedical Informatics Centre, The University of Melbourne, Melbourne, VIC, Australia

The authors have done a rigorous job of addressing the comments of the initial round of reviews. Thank you for that effort.

 To clarify my comments on word embeddings in the context of topic modelling, I wasn't actually suggesting to use external resources for word embeddings; rather, since you are already building word embeddings, to use an alternative -- potentially more effective -- strategy for producing the clusters from those word embeddings. But your modifications to address this point are fine.

I have read this submission. I believe that I have an appropriate level of expertise to confirm that it is of an acceptable scientific standard.

# Peer review: Referee response for version 2

**DOI:** 10.5256/f1000research.16078.r33788

**Published:** 2018-05-16

**Authors:** Jessica Tenenbaum, Julian Hong

**Affiliations:** 1 Department of Radiation Oncology, Duke University School of Medicine, Durham, NC, USA; 2 Department of Biostatistics and Bioinformatics, Duke University School of Medicine, Durham, NC, USA

The authors have addressed our concerns. Thank you for clarifying the method, and thanks also for the WordToVec reference!

We have read this submission. We believe that we have an appropriate level of expertise to confirm that it is of an acceptable scientific standard.

# Peer review: Referee response for version 1

**DOI:** 10.5256/f1000research.15033.r31100

**Published:** 2018-03-22

**Authors:** Karin Verspoor

**Affiliations:** 1 School of Computing and Information Systems, The University of Melbourne, Melbourne, VIC, Australia; 2 Health and Biomedical Informatics Centre, The University of Melbourne, Melbourne, VIC, Australia

The paper introduces a strategy for unsupervised analysis of a corpus of documents in order to identify terminology related to Serious Mental Illness (SMI). It applies a process of (1) identification of frequent terms (n-grams), (2) clustering of terms based of word embedding vector similarity using k-means, (3) scoring of clusters using mappings to known SMI concepts, (4) manual annotation of concepts/terms (which?) to categories. The authors then provide a detailed analysis supported by manual review by two psychiatrists. A substantial number of new relevant symptomology terms are identified through this process.

 The authors apply standard approaches/tools for doing the text analysis, which are generally well explained and easy to follow. The authors do not directly justify the frequency floor of 10, or provide details of how many of each type of n-gram (uni/bi/tri-grams) were identified in the data. There appear to be very few trigrams, for instance. Using only frequency, how do you prevent uninteresting patterns such as "of the"?

 The manual data cleaning process could have been performed semi-automatically with simple string processing tools; was this considered? (Why else would an informatician specifically need to do it?)

 The intuitions underlying the cluster scoring functions are not clearly stated; we are told it is related to prior knowledge of concepts but it is unclear what the objectives of the specific formulas presented/used are. Why are outliers of particular interest?

 For future work, the authors may be interested in experimenting with topic modeling rather than k-means clustering; see ^1^ for an approach which couples word embeddings with topic modeling. This could be more effective than k-means clustering, in particular due to the challenge of having to determine a good value for "k".

 The notion of "n-gram" is not used entirely consistently with its broader usage in the literature; usually that refers specifically to a term of a given length (e.g. 1-grams/unigrams, 2-grams/bigrams) while different length terms are mixed here. The authors might consider using the word "term", or they have referred to "concepts" which seem to be equivalent to terms.

 Regarding the limitation that no context was provided to the annotators; would it make sense to provide a concordance of some of the instances of the terms to the annotators in future efforts?

 Also, the IAA is tied to the 9 categories defined on page 7; where do these categories come from? Are they related to standard or validated frameworks for symptoms in psychiatric assessment? If not, why were those categories chosen?

 Did you perform any error analysis to explore the IAA further, e.g. a confusion matrix between categories? Is it possible that rather than considering these categories to be independent (the typical assumption for Cohen's Kappa) that some overlap between the categories might be expected?

 The data is protected by patient privacy constraints and hence cannot be made openly available (indeed the authors could only work on the data in a restricted "offline" setting). Given the nature of the data, this is understandable. OTOH, given that the analysis largely makes use of existing code plus extensions for scoring functions, it would make sense to share the *methods* in an open repository.

 The authors should include a suitable reference for PANSS. There is also a substantial literature on terminology induction (e.g. ^2^) which would be appropriate to reference.

 The writing in the manuscript is generally clear, although I identified a few things that could be rephrased or clarified:

- The word "depiction" seems to mean "usage" or "phrase" or "expression" or similar; "depiction" is typically used in the context of art or illustration and I found it strange in a language-expression related context.
- Are phenotypes and symptomatology always the same thing? Is a phenotype a *set* of behaviours/symptoms?
- The abstract is not as clear as it could be. The final sentence of the Background paragraph should use "it is" rather than "it's" but more importantly it implies that the objective is to assess clinician *preferences* as opposed to actual *usage*. Are these the same? Also, n-grams, vector space models, concepts, vocabulary and depictions are all introduced; it is a bit confusing without having read the full paper. I wonder if it could be simplified somewhat?
- As a nitpick, in the Introduction the authors refer to "predicting the diversity of vocabulary"; the work does not address *prediction* of vocabulary or its diversity but rather involves *analysis* of that vocabulary.
- Another nitpick in the Introduction is the use of the term "authorship"; I suppose the authors mean "writing" or "description" or "summary" or similar.

I have read this submission. I believe that I have an appropriate level of expertise to confirm that it is of an acceptable scientific standard, however I have significant reservations, as outlined above.

## References

[1] NguyenDQBillingsleyRDuLJohnsonM: Improving Topic Models with Latent Feature Word Representations. .2015;3:299-313

[2] FrantziKAnaniadouSMimaH: Automatic recognition of multi-word terms:. the C-value/NC-value method. .2000;3(2) : 10.1007/s007999900023 115-130 10.1007/s007999900023

# Response

**Published:** 2018-04-21

**Authors:** Richard Jackson

Thank you very much for your insightful comments.

 Please see our responses below:

*The authors apply standard approaches/tools for doing the text analysis, which are generally well explained and easy to follow. The authors do not directly justify the frequency floor of 10, or provide details of how many of each type of n-gram (uni/bi/tri-grams) were identified in the data. There appear to be very few trigrams, for instance. Using only frequency, how do you prevent uninteresting patterns such as "of the"?*

 We started this project with the explicit intention of making as few assumptions as possible about semantic relationships contained within the 20 million documents in the CRIS corpus. To this end, we kept pre-processing very light, and no attempt was made to eliminate very common n-grams. Our chief assumption in planning the methodology was that uninteresting, high frequency n-grams that appear in many contexts would occupy locations in the vector space a substantial distance away from the n-grams we were interested in (symptomatology). We therefore sought to maximise the performance of our clustering method and scoring algorithm, which we hoped would filter off the n-grams that carry little information. In addition, any uninteresting patterns that did survive the filter, we removed via some simple string processing tools (see response to your additional question below on this). Regarding the counts of different n-grams, we have added the following to the vocabulary creation subsection:

 “

 Sentences and tokens were extracted from each document using the English Punkt tokeniser from the NLTK 3.0 suite\cite{bird_natural_2009}. Each token was converted to lower case. A vocabulary was then constructed of all 1-gram types in the corpus, supplemented with frequently occuring bi-grams and tri-grams using the Gensim\cite{rehurek_lrec} suite and the sampling method proposed by Mikolov \textit{et al}\cite{mikolov2013distributed}. Bi-grams and tri-grams with a minimum frequency of 10 occurrences in the entire corpus were retained, to give a total vocabulary size of 896 195 terms (617 095 unigrams, 277 490 bigrams, 303 trigrams and 1307 non-word entities). No further assumptions about the structure of the data, such as the need for stemming/lemmatisation, were made.

 ”

*The manual data cleaning process could have been performed semi-automatically with simple string processing tools; was this considered? (Why else would an informatician specifically need to do it?)*

 Indeed it was, although this was not made clear in the manuscript. We have added a short piece of text (in the “expert curation” subsection) to explain this:

 “

 The contents of the top scoring clusters underwent a two stage curation process. The first stage was performed by an informatician, and involved several simple string processing tasks to filter out uninteresting n-grams. Such processes included removal of n-grams that contained tokenisation failures (for example, single character non-word tokens such as ‘y’, ‘p’) and other constructs that had low information content, such as n-grams composed of stop words. A final manual check followed to reduce the amount of annotator burden required by the clinical team.

 ”

*The intuitions underlying the cluster scoring functions are not clearly stated; we are told it is related to prior knowledge of concepts but it is unclear what the objectives of the specific formulas presented/used are. Why are outliers of particular interest?*

 We accept this was not clearly stated and have adjusted the manuscript accordingly, in the “Vocabulary clustering and cluster scoring” section:

 “

 With the data clustered, we sought to identify one or more clusters of interest for further examination. To this end, we devised a simple `relevance' cluster scoring approach based upon prior knowledge of common SMI symptom concepts. The intuition behind our approach is that the training of the Word2Vec model will cause n-grams that represent `known' concepts of SMI symptomatology to co-locate in close proximity to each other in vector space, and the clustering approach will place them in the same cluster, along with other n-grams that theoretically relate to these SMI symptomatology concepts. The additional contents of this cluster may therefore hold n-grams that represent concepts of SMI symptomatology undefined by our team, but in natural use by the wider clinical staff of the SLAM Trust during the course of their duties. By identifying the richest cluster(s) in terms of the known SMI symptomatology lexicon, we sought to drastically reduce the search space of n-grams in the corpus to carry forward for human assessment.

 ”

*For future work, the authors may be interested in experimenting with topic modeling rather than k-means clustering; see 1 for an approach which couples word embeddings with topic modeling. This could be more effective than k-means clustering, in particular due to the challenge of having to determine a good value for "k".*

 We agree that recent advancements in topic modelling approaches are relevant to our work here. Regarding the specific case of using external word embedding models, we suspect that our target domain, (UK clinical text), is a sub-language, and the use of external word embeddings (even from very large corpora, such as Google News in the model proposed in the suggested citation) will have limited value for discovery on our data. The concepts of interest in our work are technical in nature, and seem likely to be specific to heavily regulated documents and therefore unlikely to exist in publically available datasets. On the other hand, there is no requirement to build the word embedding model from external datasets. Ultimately, there’s clearly a range of additional techniques in the literature that would be worthwhile experimenting with. We have updated our discussion as follows:

 “

 During peer review, it was suggested that recent advancements in topic modelling approaches may be relevant to our work. Many groups have sought to combine the popular technique of Latent Dirichlet Allocation (LDA)\cite{blei2003latent} with word embedding models to derive appropriate terminology for a given topic\cite{cao2015novel,hinton2009replicated,srivastava2013modeling}. For instance, Nguyen et al\cite{nguyen2015improving} propose an extension of LDA that makes use of a word embedding model trained on a very large corpus of text to improve the performance of topic coherence modelling on several datasets. Future work might seek to explore such techniques, and (assuming regulatory barriers can be overcome), the potential of creating word embedding models from very large clinical text corpora by combining data with other care organisations.

 “

*The notion of "n-gram" is not used entirely consistently with its broader usage in the literature; usually that refers specifically to a term of a given length (e.g. 1-grams/unigrams, 2-grams/bigrams) while different length terms are mixed here. The authors might consider using the word "term", or they have referred to "concepts" which seem to be equivalent to terms.*

 For technical clarity, we’ve removed references to ‘n-gram’ and replaced them with ‘term’ where appropriate. Our usage of concept refers to medical concepts (via our putative discovery process or otherwise). This is now consistent in our amendments.

 Regarding the limitation that no context was provided to the annotators; would it make sense to provide a concordance of some of the instances of the terms to the annotators in future efforts?

 We agree this would be a useful method to assist in the decision making process for manual curation, and have adjusted the text:

 “

 The results of our IAA exercise between two experienced psychiatrists suggested a moderate level of agreement in categorising the newly identified constructs. Given that this annotation exercise did not provide any context beyond the n-gram, and that the nature of SMI symptom observation is somewhat subjective, perhaps it is to be expected that agreement was not higher. As suggested during peer review, providing a concordance of some of the instances of each n-gram, along with expert panel discussion and engagement with international collaborative efforts in SMI research may prove valuable in seeking more formal definitions of the identified constructs.

 ”

*Also, the IAA is tied to the 9 categories defined on page 7; where do these categories come from? Are they related to standard or validated frameworks for symptoms in psychiatric assessment? If not, why were those categories chosen?*

 The categories were derived from the Shorter Oxford Textbook of Psychiatry (chapter 3, page 44), and the experience of the teams Clinical Psychiatrists. We have updated the text as follows:

 “

 The second, more important stage was composed of independent annotation of the curated concept list by two psychiatrists, to identify likely synonyms and new symptomatology based on their clinical experience. Each concept was assigned to one of the below 8 `substantive' categories, or a 9th `other' category. The categories were derived from\cite{harrison_shorter_2018}, and the experience of the team Clinical Psychiatrists.

 ”

*Did you perform any error analysis to explore the IAA further, e.g. a confusion matrix between categories? Is it possible that rather than considering these categories to be independent (the typical assumption for Cohen's Kappa) that some overlap between the categories might be expected?*

 No further attempts to explore the errors in IAA were made in this analysis. Given the high level of cross-sectional and longitudinal overlap between mental disorder diagnoses classified as ‘SMI’ and the subjectivity involved in observation, it’s reasonable to think that there would be a tendency for errors to overlap in certain categories (for instance ‘insight’ and ‘cognition’). However, we think that this is outweighed by the far more complex issue of the clinical validation of the concepts we identified (which the scope of this study did not allow for).

*The data is protected by patient privacy constraints and hence cannot be made openly available (indeed the authors could only work on the data in a restricted "offline" setting). Given the nature of the data, this is understandable. OTOH, given that the analysis largely makes use of existing code plus extensions for scoring functions, it would make sense to share the methods in an open repository.*

 We agree this would be useful, and now provide a link to a repository in the paper:

 “

 Example code used in this analysis is available at: https://github.com/RichJackson/clustering_w2v

 ”

*The authors should include a suitable reference for PANSS. There is also a substantial literature on terminology induction (e.g. 2) which would be appropriate to reference.*

 We think that the reviewer might have missed our original reference to the PANSS on page 3. However we mistakenly repeated the full acronym on page 10. This is now corrected.

 Regarding terminology induction, we have modified  the following text in the introduction as follows. This now includes a reference to this article which we feel is particularly topical, given our domain:

 "

 First, insight must be obtained regarding real-world language usage such that universally understood medical entities, encompassing hypernomy, synonymy and hyponomy adequately represent models of concepts. Similarly, because of the abundant use of acronyms in the medical domain, a large percentage have two or more meanings\cite{liu_study_2002}, creating word sense disambiguation problems. As such, significant efforts have arisen to supplement these types of knowledge bases with appropriate real world synonym usage extracted from EHR datasets\cite{henriksson_identifying_2013}. The problem may be considered analogous to difficulties in the recognition, classification and mapping of technical terminology variants throughout the biomedical literature, which is known to be an impediment to the construction of knowledge representation systems (see \cite{krauthammer_term_2004} for a review).

 Krauthammer, Michael, and Goran Nenadic. "Term identification in the biomedical literature." Journal of biomedical informatics 37.6 (2004): 512-526.

 "

*The writing in the manuscript is generally clear, although I identified a few things that could be rephrased or clarified:*

*The word "depiction" seems to mean "usage" or "phrase" or "expression" or similar; "depiction" is typically used in the context of art or illustration and I found it strange in a language-expression related context.*

 We have rephrased this language throughout

*Are phenotypes and symptomatology always the same thing? Is a phenotype a set of behaviours/symptoms?*

 Yes - the definition of a phenotype is the set of observable characteristics of an organism resulting from its genotype and interaction with its environment. We believe that symptom/behaviour profiles can be reasonably viewed in this way (and these are commonly referred to in phenotypic terms in mental health research).

*The abstract is not as clear as it could be. The final sentence of the Background paragraph should use "it is" rather than "it's" but more importantly it implies that the objective is to assess clinician preferences as opposed to actual usage. Are these the same?*

 You are correct to say that ‘actual usage’ and ‘clinical preference’ are different concepts here. Our work aims to capture ‘preference’ in clinical language constructs that do not reflect matches to industry knowledge base projects (regardless of the reason). We have made several small changes in the text to reflect this.

 Also, n-grams, vector space models, concepts, vocabulary and depictions are all introduced; it is a bit confusing without having read the full paper. I wonder if it could be simplified somewhat?

 This now reads:

 “

 By utilising a large corpus of healthcare data, we sought to make use of semantic modelling and clustering techniques to represent the relationship between the clinical vocabulary of internationally recognised SMI symptoms and the preferred language used by clinicians within a care setting. We explore how such models can be used for discovering novel vocabulary relevant to the task of phenotyping Serious Mental Illness (SMI) with only a small amount of prior knowledge.

 “

*As a nitpick, in the Introduction the authors refer to "predicting the diversity of vocabulary"; the work does not address prediction of vocabulary or its diversity but rather involves analysis of that vocabulary.*

 This sentence now reads:

 “

 Given a sufficiently large corpus of documents, typically authored by hundreds of clinical staff over several years, it is often difficult to track the evolution of vocabulary used within the local EHR setting to describe potentially important clinical constructs.

 ”

*Another nitpick in the Introduction is the use of the term "authorship"; I suppose the authors mean "writing" or "description" or "summary" or similar.*

 We’ve also addressed this.

 Thanks once again for your valuable insights

# Peer review: Referee response for version 1

**DOI:** 10.5256/f1000research.15033.r31101

**Published:** 2018-03-21

**Authors:** Jessica Tenenbaum, Julian Hong

**Affiliations:** 1 Department of Radiation Oncology, Duke University School of Medicine, Durham, NC, USA; 2 Department of Biostatistics and Bioinformatics, Duke University School of Medicine, Durham, NC, USA

The authors present a method to extract from a clinical corpus novel terms used to described serious mental illness (SMI). They use vector space models to represent the relationship between words in the corpus and combine this approach with clustering techniques and manual curation to identify relevant n-grams (1, 2, or 3-word concepts). 106 concepts had no mapping to current SNOMED terms indicating that they have indeed discovered new knowledge, i.e. terms used by clinicians to describe patients that are not already included in SNOMED CT.

  

1. The introduction was unusually well written, if a little longer than strictly necessary, and provided excellent motivation for the work at hand. It has been shown that SNOMED coverage of mental health terms is sub-optimal and this is a clever approach to learning new relevant terms in a semi-automated manner.
2. For the rest of the paper, each individual part was well written, but I had a hard time seeing how they flowed together.
3. Figure 1 was a helpful overview, but I still found it difficult to follow how the sub-steps tied in together and in some cases why they were important. e.g.
  1. How did creation of the putative cluster of 38 terms help? I think it was to facilitate the scoring method, but I wasn't completely clear how.
  2. Why 38? Particularly when the clusters they later looked at were so much larger, not clear why that number was chosen.
4. I found the math/logic challenging to follow. (Admittedly, I am not a statistician, and was not previously familiar with CBOW.)
  1. It was helpful that the authors included examples in some places, but they could have gone even further to make the approach concrete. Toy examples of u1 and v1 would help.
  2. On first and second read, I was having a hard time with intuition for what a high-scoring cluster means. I now realize (I think?) it meant the cluster was particularly enriched for mental health terms. It might be helpful to state that- for some reason I was thinking it meant that the concepts were relatively similar/cohesive?
  3. I had trouble wrapping my head around the sentence "we scored each cluster based on the number of per concept hits to derive a cluster/concept count matrix x where xi,j represents the count of the ith concept in the jth cluster." I think it means 38 rows, 1 for each concept and 3 columns, 1 for each cluster, and the value of the cell is the number of times that concept was encountered  in some form in the cluster?
  4. Equations could also be numbered for reference.
5. The authors report choosing not to perform stemming/lemmatization in order not to make assumptions about the structure of the data, but this decision is not very well explained or justified. Indeed they call it out as a potential limitation in Discussion. It would be useful/interesting to try the approach both ways and see if the results were different.
6. How were the 8 "substantive categories" chosen?
7. Why does inter-rater agreement matter in mapping the concepts to those categories? Was it only that the ability to map them to a single category makes it more likely the concept is semantically interesting and reliable?
8. The authors mention that the semantic similarity of n-grams is often measured via their cosine distance between vectors in the W matrix. Just out of curiosity, could distance in the W' matrix be used as well/instead?
9. My "partly" answer to "Are sufficient details of methods and analysis provided to allow replication by others?" reflects the fact that the authors very reasonably cannot publish the raw data, but they do address how to obtain the data through a formal application process. (Ergo the "Yes" to whether source data are available, even if not readily…) It would be helpful if code were to be made available.

 

 Minor:

1. Page 3, paragraph 4 should be employS curated terminology
2. Page 6 line 5 should have ) after "Table 1"

We have read this submission. We believe that we have an appropriate level of expertise to confirm that it is of an acceptable scientific standard, however we have significant reservations, as outlined above.

# Response

**Published:** 2018-04-21

**Authors:** Richard Jackson

Thank you for your valuable comments. Please see our responses below

*For the rest of the paper, each individual part was well written, but I had a hard time seeing how they flowed together.*

*Figure 1 was a helpful overview, but I still found it difficult to follow how the sub-steps tied in together and in some cases why they were important. e.g.*

*How did creation of the putative cluster of 38 terms help? I think it was to facilitate the scoring method, but I wasn't completely clear how.*

*Why 38? Particularly when the clusters they later looked at were so much larger, not clear why that number was chosen.*

 We think there’s some misunderstanding of our methodology and apologise if it wasn’t clear in the manuscript. We didn’t create any clusters in this work by hand. All of the 896 195 terms generated from the corpus were assigned to one of 75  distinct clusters via the K-means algorithm. We then needed to identify which of the 75 clusters were worth looking at. The 38 concepts constitute the prior knowledge about SMI symptomatology that our clinical team fed into the scoring algorithm we describe in the manuscript. This revealed three clusters that we took forward for further analysis.

 We’ve re-written the text concerning this, and introduced something we call ‘Prior Concepts’ to differentiate between the domain knowledge we use in cluster scoring and the clusters themselves. Please do let us know if you feel that this hasn't improved the manuscript clarity.

*I found the math/logic challenging to follow. (Admittedly, I am not a statistician, and was not previously familiar with CBOW.)*

*It was helpful that the authors included examples in some places, but they could have gone even further to make the approach concrete. Toy examples of u1 and v1 would help.*

*We’ve added an example of the analysis pipeline to the accompanying code repository, and added the line:*

 “

 We provide a worked example of this technique in the code repository that accompanies this paper, using publically available data.

 “

*On first and second read, I was having a hard time with intuition for what a high-scoring cluster means. I now realize (I think?) it meant the cluster was particularly enriched for mental health terms. It might be helpful to state that- for some reason I was thinking it meant that the concepts were relatively similar/cohesive?*

 Actually you are correct on both counts. Training the Word2Vec model causes n-grams with semantically similar meanings to co-locate near to each other in the vector space model. The application of the clustering algorithm groups semantically similar n-grams together. Three clusters scored highly in our relevancy scoring algorithm, signifying that they were enriched for our existing  knowledge of SMI symptomatology.

*I had trouble wrapping my head around the sentence "we scored each cluster based on the number of per concept hits to derive a cluster/concept count matrix x where xi,j represents the count of the ith concept in the jth cluster." I think it means 38 rows, 1 for each concept and 3 columns, 1 for each cluster, and the value of the cell is the number of times that concept was encountered  in some form in the cluster?*

 This is almost correct, although j represents the total number of clusters (75). The result is a score per cluster that is plotted in figure 3. We have rewritten this section of text accordingly, as per the previous comment on this issue

*Equations could also be numbered for reference.*

 This is now done

*The authors report choosing not to perform stemming/lemmatization in order not to make assumptions about the structure of the data, but this decision is not very well explained or justified. Indeed they call it out as a potential limitation in Discussion. It would be useful/interesting to try the approach both ways and see if the results were different.*

 This is a potential limitation of our work, in that using un-stemmed tokens will have led to vastly more n-grams than we might have otherwise had to deal with, and that stemming might have lead to the identification of additional n-grams of interest. However, we feel our decision not to make assumptions about the value of stemming in this context was appropriate for two reasons:

1. In the context of a mental health assessment, stemming may cause important information loss. For instance, the term ‘insomnia’ shares the same stem as ‘insomniac’. However, short term ‘insomnia’ is a relatively common symptom amongst the general population for a large variety of conditions. ‘Insomniac’ in the context of mental illness, on the other hand,  might imply a chronic condition.
2. Our IAA task would have been substantially more complex if we were to offer stemmed n-grams for human evaluation, rather than complete words.

*How were the 8 "substantive categories" chosen?*

 The categories were derived from the Shorter Oxford Textbook of Psychiatry (chapter 3, page 44), and the experience of the teams Clinical Psychiatrists. We have updated the text as follows:

 “

 The second, more important stage was composed of independent annotation of the curated concept list by two psychiatrists, to identify likely synonyms and new symptomatology based on their clinical experience. Each concept was assigned to one of the below 8 `substantive' categories, or a 9th `other' category. The categories were derived from\cite{harrison_shorter_2018}, and the experience of the team Clinical Psychiatrists.

 ”

*Why does inter-rater agreement matter in mapping the concepts to those categories? Was it only that the ability to map them to a single category makes it more likely the concept is semantically interesting and reliable?*

 Yes, we felt that offering a binary choice per n-gram (i.e. potentially relevant/irrelevant) was likely to heavily bias our results in favour of high agreement. Rather, we thought that agreement on the mapping of the identified n-grams to defined groups of symptomatology would suggest a greater degree of robustness.

*The authors mention that the semantic similarity of n-grams is often measured via their cosine distance between vectors in the W matrix. Just out of curiosity, could distance in the W' matrix be used as well/instead?*

 We don’t believe this is possible, as the W matrix corresponds to weights between the input layer and the hidden layer of the neural network, where each row represents a single n-gram. The W' corresponds to the weights between the hidden layer and the output layer, which has different dimensions.  I recommend this reference for a detailed description of the Word2Vec methodology.

*My "partly" answer to "Are sufficient details of methods and analysis provided to allow replication by others?" reflects the fact that the authors very reasonably cannot publish the raw data, but they do address how to obtain the data through a formal application process. (Ergo the "Yes" to whether source data are available, even if not readily…) It would be helpful if code were to be made available.*

 We agree this would be useful, and now provide a link to a repository in the paper:

 “

 Example code used in this analysis is available at: https://github.com/RichJackson/clustering_w2v

 ”

*Minor:*

*Page 3, paragraph 4 should be employS curated terminology*

*Page 6 line 5 should have ) after "Table 1*

 This is now corrected.

 Thanks once again for your time and efforts with our paper.

 Best wishes

 Richard
